# Gold nanoparticles with patterned surface monolayers for nanomedicine: current perspectives

**DOI:** 10.1007/s00249-017-1250-6

**Published:** 2017-09-01

**Authors:** Paolo Pengo, Maria Şologan, Lucia Pasquato, Filomena Guida, Sabrina Pacor, Alessandro Tossi, Francesco Stellacci, Domenico Marson, Silvia Boccardo, Sabrina Pricl, Paola Posocco

**Affiliations:** 1 Department of Chemical and Pharmaceutical Sciences, INSTM Trieste Research Unit, University of Trieste, 34127 Trieste, Italy; 2 Department of Architecture and Engineering (DEA), University of Trieste, 34127 Trieste, Italy; 3 Department of Life Sciences, University of Trieste, 34127 Trieste, Italy; 4 Institute of Materials, École Polytechnique Fédérale de Lausanne, 1015 Lausanne, Switzerland; 5 Molecular Simulation Engineering Laboratory (MOSE), DEA and INSTM Research Unit MOSE-DEA, University of Trieste, 34127 Trieste, Italy

**Keywords:** Mixed self-assembled monolayers, Nano-bio interface, Molecular modeling, Membrane penetration, Patchy gold nanoparticles, Cells

## Abstract

Molecular self-assembly is a topic attracting intense scientific interest. Various strategies have been developed for construction of molecular aggregates with rationally designed properties, geometries, and dimensions that promise to provide solutions to both theoretical and practical problems in areas such as drug delivery, medical diagnostics, and biosensors, to name but a few. In this respect, gold nanoparticles covered with self-assembled monolayers presenting nanoscale surface patterns—typically patched, striped or Janus-like domains—represent an emerging field. These systems are particularly intriguing for use in bio-nanotechnology applications, as presence of such monolayers with three-dimensional (3D) morphology provides nanoparticles with surface-dependent properties that, in turn, affect their biological behavior. Comprehensive understanding of the physicochemical interactions occurring at the interface between these versatile nanomaterials and biological systems is therefore crucial to fully exploit their potential. This review aims to explore the current state of development of such patterned, self-assembled monolayer-protected gold nanoparticles, through step-by-step analysis of their conceptual design, synthetic procedures, predicted and determined surface characteristics, interactions with and performance in biological environments, and experimental and computational methods currently employed for their investigation.

## Introduction

Use of nanomaterials (NMs) for biomedical applications is a rapidly growing research field, thanks to enormous progress in recent decades in manipulating materials down to the nanoscale. Active work has been done in developing NMs not only as diagnostic or therapeutic agents, but also as smart nanoplatforms with maximized biospecificities (Blanco et al. 2015; Chen et al. 2016; Chou et al. 2011; Mahon et al. 2012; Ryu et al. 2014; Wolfram et al. 2015). Despite rapid advances in preparation of these sophisticated nanosystems, the keys to rational design required to control their biointeractions remain elusive. The reason lies mainly in the complexity of the problem; biological matrices are multicomponent systems in which interactions taking place at cellular and subcellular levels are far from being simply additive. At the same time, NMs come in a variety of shapes, sizes, and surface chemistries, all of which may affect their bioactivity. Interfacing NMs with biological systems thus adds complexity to complexity, making it difficult to probe the combined system adequately using existing techniques.

Engineered gold nanoparticles (AuNPs), particularly when surface-modified with self-assembled monolayers (SAMs), are excellent tools to elucidate fundamental properties of biological interactions at the nano- and molecular scales. Interest in SAM-protected AuNPs is motivated by the relatively inert and therefore biocompatible nature of gold, by their specific surface chemistry, and by their unique electronic and optical properties, together with the availability of a convenient range of processing technologies (Alkilany et al. 2013; Dreaden et al. 2012; Li et al. 2014a; Love et al. 2005). Broadly speaking, SAMs are organic assemblies formed by adsorption of molecular constituents onto flat or curved solid surfaces. The ability to precisely control the composition of the monolayer has made it possible to examine structure–property relationships and to synthesize well-defined organic surfaces with tailored molecular and macroscopic properties (Love et al. 2005).

### Mixed self-assembled monolayers on gold nanoparticles

**Fig. 1:**
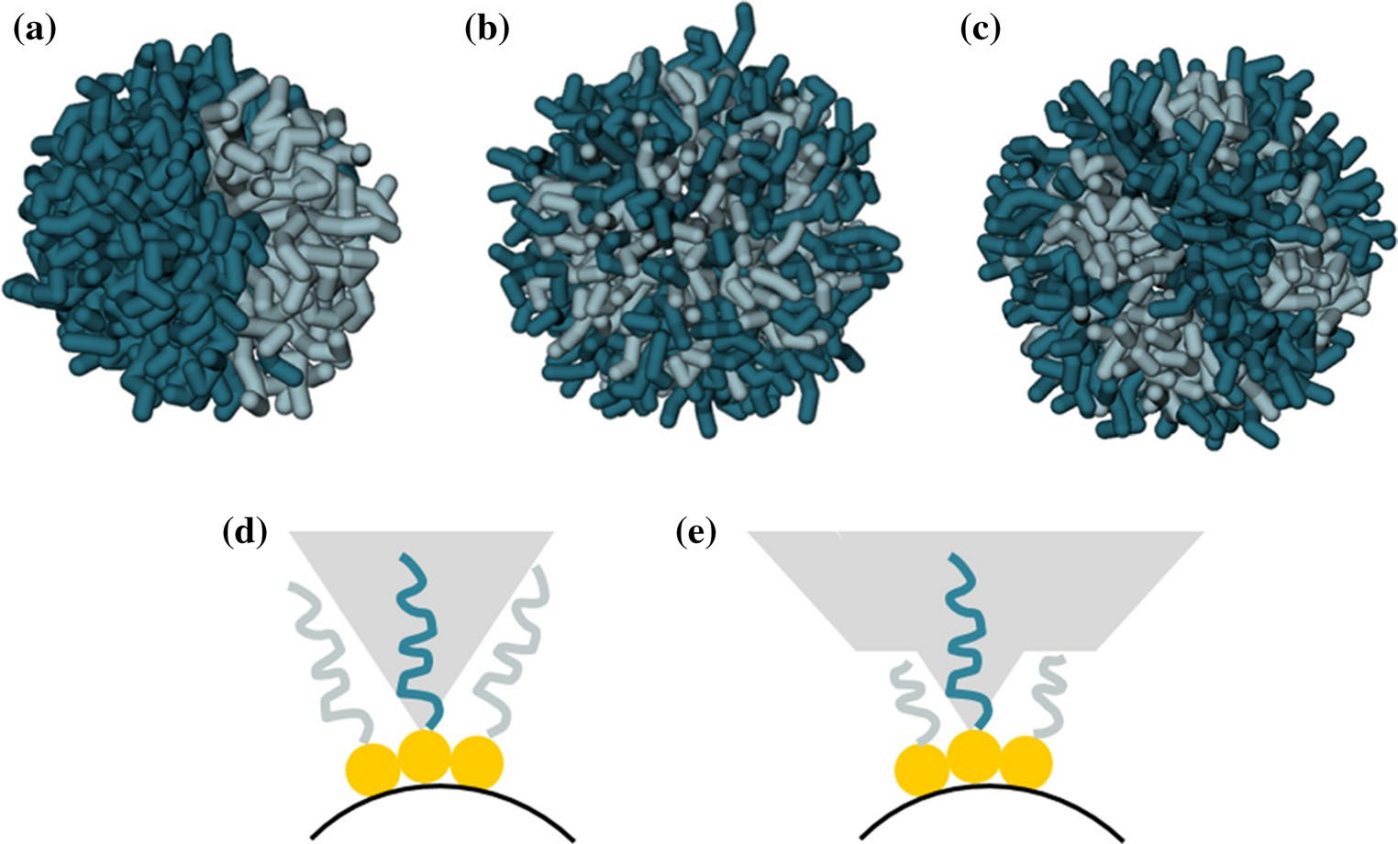
Typical 3D organization of two immiscible ligands (*dark-* and *light-blue sticks*) on a curved surface: Janus (**a**), mixed random (**b**), and regularly striped (**c**). Schematic representation of the free volume (*grey area*) that the ligand tails are allowed to sample on an NP surface, when surrounded by other types of surfactant chains of the same (**d**) or different length (**e**) on curved surfaces. Ligand length mismatch endows longer tails with more available free volume, which results in an interface entropy gain and favors striped pattern formation over complete phase segregation

When mixed SAMs of unlike ligand molecules are employed to coat AuNPs, nanoscale domains tend to form spontaneously within the surface ligand shell. The formation of 3D patterns (typically patched, striped or Janus-like domains; Fig. 1) is dictated by the competition between two effects: energy minimization, which tends to reduce contact between immiscible surfactants, and maximization of conformational entropy, gained by forming extended interfaces between surfactants of different length or bulkiness. While only a few limiting morphologies are possible when a binary mixture of ligands is used, their number increases considerably when more complex ligand combinations are considered (Pons-Siepermann and Glotzer 2012a, b). This peculiarity is intriguing, as it provides access to a diversity of possible patterns and allows tuning of their morphological characteristics on the basis of easy-to-control parameters, e.g., surfactant length (Ghorai and Glotzer 2010; Jackson et al. 2004; Singh et al. 2007) or NP radius (Carney et al. 2008; DeVries et al. 2007; Devries et al. 2008; Kim et al. 2012), as well as less governable parameters such as the degree of immiscibility (Ge et al. 2015; Gentilini et al. 2009; Krafft and Riess 2009; Posocco et al. 2012) and stoichiometry of the SAM components (Şologan et al. 2016b) (see below for further discussion on the parameters driving self-sorting of ligand mixtures).

Presence of discrete domains at the nanometer level bestows surface properties (such as interfacial energy, solubility, or wettability) to engineered AuNPs that cannot be explained simply based on the bulk composition alone (Centrone et al. 2008; Kuna et al. 2009), and allows tailoring of nanosurfaces with a wide array of specific features. Furthermore, as proteins and other biological structures themselves exhibit nanoscale patterning of hydrophilic/hydrophobic groups, such nanoscale heterogeneity of SAM-protected NPs is expected to have significant implications for surface-related biological processes such as protein adsorption (Huang et al. 2013, 2014; Hung et al. 2011, 2013) and membrane interaction (Carney et al. 2012; Sabella et al. 2014; Van Lehn and Alexander-Katz 2014a, 2015; Van Lehn et al. 2013, 2014; Verma et al. 2008).

Another attractive feature of patterned NPs is that they are subject to directional interactions, which promote controlled self-assembly into complex yet predictable 3D structures (DeVries et al. 2007; Moffitt 2013; Zhang et al. 2012). SAM-modified AuNPs may thus be envisaged as remarkably versatile precursors to a wide variety of patchy-particle building blocks.

### Mixed SAM-functionalized gold nanoparticles in nanomedicine

In the light of the above considerations, intensive studies have been carried out on the effects of various properties of monolayer-protected gold nanoparticles (MPNPs) on their behavior in a physiological environment (Mu et al. 2014). Surface modification of MPNPs has thus emerged as an essential tool to modulate the behavior of these materials both in vitro and in vivo (Feliu et al. 2016; Jiang et al. 2015; Sabella et al. 2014; Tay et al. 2014). In this respect, several essential aspects of NPs, such as their biodistribution, toxicity, clearance, and cellular uptake, can be regulated through controlled physicochemical surface modifications (Beddoes et al. 2015). In particular, the relative spatial arrangement of ligands on the AuNP surface plays a key role in mediating their cellular uptake (Carney et al. 2012; Jewell et al. 2011; Sabella et al. 2014; Verma et al. 2008).

Another important facet of NPs for bioapplications is their fate when exposed to biological fluids such as plasma or serum. Circulating proteins (or other biomolecules) can readily adsorb onto their surface, forming a so-called protein corona (Lazarovits et al. 2015; Maiolo et al. 2015). The corona confers a “biological identity” on NPs that adds to their “synthetic” identity and has a determining effect on NP–cell interactions. In this respect, surface morphology is a leading actor, as striped NPs are reported to be significantly more effective at avoiding nonspecific adsorption of a variety of proteins compared with other morphologies due to the unique alternating distribution of hydrophobic and hydrophilic regions on their surface on a subnanometer scale (Jackson et al. 2004).

The fate of a MPNP in a biological environment therefore depends on both the nature of its engineered surface and the multiple biological components it encounters (proteins, cell membranes, polysaccharides, DNA, etc.). Interactions at the nano–bio interface are governed by the interplay of—and sometimes competition between—multiple chemical and physical interactions acting at different dimensions and energy scales. The overall outcome is quite difficult to predict a priori, as this requires deep understanding of the dynamic forces and molecular characteristics that shape all these interactions (Murphy et al. 2015).

Although a thorough description of the main bio–physicochemical interactions acting at the nano–bio interface of MPNPs with a biological system remains an open challenge, rational design approaches for recognizing, at least in part, how the properties of engineered NPs relate to their biological behavior could be established in principle. This requires integration of complementary experimental and theoretical methodologies, ranging from synthetic strategies for directing the formation of the patterned surfaces, to physical methods for judging the resulting structures, biochemical/biological procedures for analyzing biointeractions and NP effects, as well as ad hoc computational methodologies for modeling both NP structures and biointeractions at molecular resolution.

In this scenario, AuNPs protected by nanoscale patterned SAMs represent ideal substrates for these studies; accordingly, we begin this review by rationalizing the principles governing the self-sorting of ligand mixtures on the metal surface per se, on the basis of both theoretical and experimental evidence. Particular attention is devoted to those in silico/in vitro techniques needed to synthesize and characterize the patterned monolayer morphology. Next, we outline how different SAM organizations mediate progressively more complicated biological interactions, from model membranes or discrete proteins to living cells.

## How spontaneous ligand organization generates nanoscale complexity

Spontaneous patterning of mixed monolayers requires self-sorting of the constituent units in the absence of an external template. Understanding the properties responsible for this auto-organization is essential for design of systems with well-defined and controlled morphologies.

**Fig. 2:**
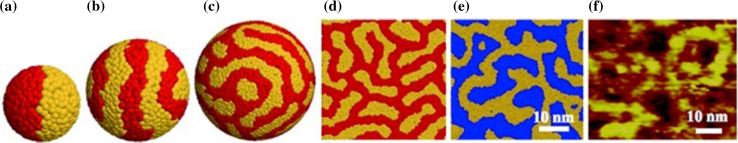
Mesoscale equilibrium structures of self-assembled organization of a binary mixture of surfactants (**a**–**d**). Ligands with length ratio C4:C7 [HS-(CH_2_)_3_-CH_3_ versus HS-(CH_2_)_6_-CH_3_] on surfaces with decreasing degree of curvature from **a** to **d**. *Red* and *yellow beads* represent the head groups of shorter- and longer-chain surfactants (*tails* not shown), respectively. Sphere radii are not drawn to scale. **e** Atomistic simulation of a C4:C6 mixed monolayer (both chains having –CH_3_ tail end-groups) showing stripe-like domains. The head groups of the short and long surfactant molecules are represented by *blue* and *yellow beads*, respectively. **f** STM height images of C4:C6 mixed monolayer showing the stripe-like morphology with domain width of ~5 nm. [Reprinted with permission from (Singh et al. 2007). Copyright (2007) The American Physical Society]

In their pioneering work, Glotzer and coworkers revealed the origin of experimentally observed stripe-like patterns formed by two immiscible ligands coadsorbed on the curved surface of AuNPs by atomistic and mesoscale simulations (Ghorai and Glotzer 2010; Jackson et al. 2004; Singh et al. 2007; Fig. 2). The stabilization of stripe-like domains was found to depend on the balance between the enthalpy of phase separation (driving macrophase separation) and conformational entropy (leading to increased ligand–ligand interface area). Conformational entropy gain may derive either from the length mismatch of dissimilar ligands, or from their different bulkiness, as schematically illustrated in Fig. 1d, e.

**Fig. 3:**
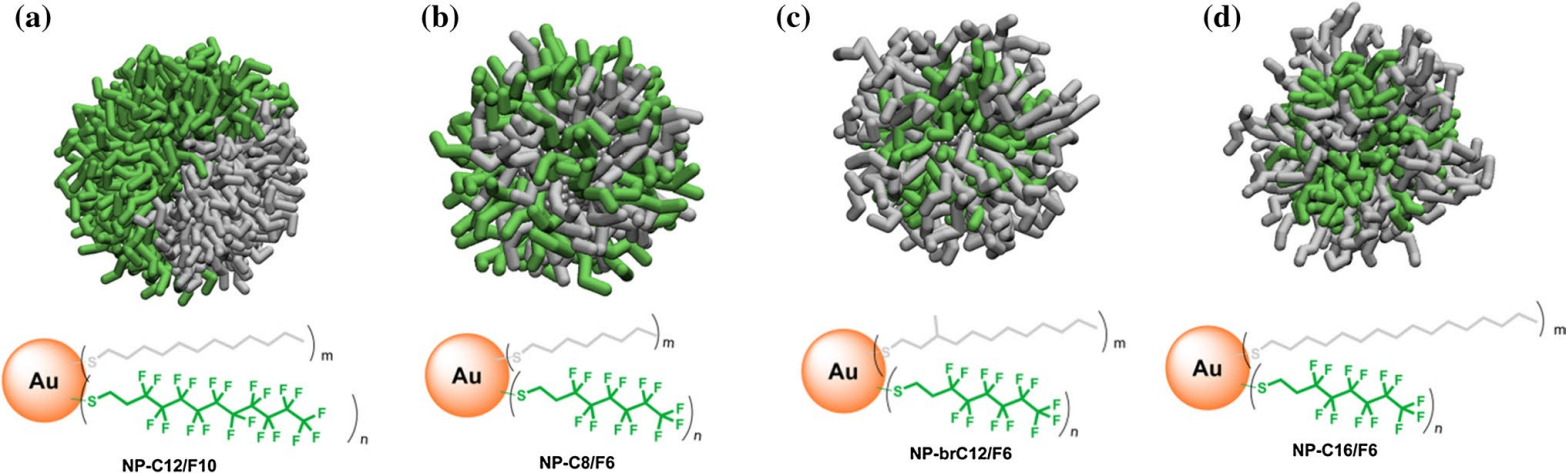
Patterns stemming from self-sorting of equimolar blends of different fluorinated (F) and hydrogenated carbon (H) thiols on surface of gold NPs. Equilibrium morphologies were predicted from CG simulations in explicit solvent. Note that C8, C12, and C16 refer to the full length of C ligand alkane chains, while F6 and F10 refer only to the number of fluorinated carbons in F thiols, with the full chains being 8 and 12 carbons in length, respectively; *m* and *n* indicate the number of F and C chains in the monolayer, respectively. Solvent omitted for sake of clarity. Color code: *gray*, H ligands; *green*,  F ligands. [Adapted with permission from (Şologan et al. 2016b). Copyright (2016) American Chemical Society]

When long or bulky ligands are adjacent to shorter or less bulky ones, the system gains additional conformational entropy by virtue of the extra free volume available to the former surfactants (Fig. 1e). If this gain in entropy is sufficient to overcome the enthalpic loss (which depends on the overall chain lengths and length mismatch relative to the surface curvature), then microphase-separated stripes, rather than bulk phase separation, occur at equilibrium. As a result of this fine enthalpy–entropy interplay, mixtures of immiscible ligands may adopt (1) a two-faced Janus morphology (Fig. 1a) when the entropy contributions are small (ligands with similar length/bulk), (2) a “random” mixed arrangement when enthalpy is small, or if an ordered arrangement is hindered by, e.g., a branched ligand structure (Liu et al. 2012) (Fig. 1b), and (3) a “striped” (or locally ordered patchy) arrangement when the two components are balanced (Fig. 1c). Stripe thickness can be tuned by tailoring the tail length of individual ligands or, in case of ionic surfactants, altering the charges on the ligand head groups (Ghorai and Glotzer 2010).

Another crucial parameter that determines the formation of ordered ripples in a SAM is the NP core size, as this affects the surface curvature. On small NPs, a binary mixture of surfactants tends to separate into two distinct phases (i.e., Janus arrangement), as all chains enjoy sufficient free volume due to the high surface curvature and there is no entropic gain on formation of new interfaces. Decreasing the surface curvature (larger NP radius) drives phase separation into ordered ripples, as the available free volume decreases and the entropic gain associated with new interfaces on “stripe” formation increases. The balance leads to a total free energy lower than that achievable by complete demixing. When the NP radius is further increased, disordered stripes and patchy domains are expected to dominate.

From a theoretical standpoint, the formation of stripe-like domains should occur in a relatively narrow NP size regime. This was indeed quantified in the 2.5–8.0 nm range (Carney et al. 2008), and confirmed via the peculiar chemical reactivity of these systems (DeVries et al. 2007; Devries et al. 2008). The existence of Janus NPs was also confirmed by experiments on gold NPs with chemically different combinations of ligands (Kim et al. 2012). For the specific systems examined, Janus morphologies formed when the core diameter was less than 1.5 nm, while a transition from Janus to striped NPs was observed in the 1.5–3.0 nm range.

Recently, atomistic discrete molecular dynamics simulations have enriched this picture by suggesting three different SAM classes, depending on whether and under what conditions striped patterns arise (Ge et al. 2015). These studies considered that, aside from entropic considerations, stripe formation can also depend on interligand interactions that emerge only for a specific subset of immiscible binary SAM systems. Immiscibility of ligands is therefore a further driving force triggering the formation of phase-separated domains, and in this respect one can exploit, for example, the reciprocal phobicity of hydrocarbon and fluorocarbon chains (Krafft and Riess 2009).

Along this line, Pasquato and coworkers combined approaches for preparing MPNPs by using mixtures of amphiphilic fluorinated and amphiphilic hydrogenated thiols bearing polyethylene glycol units of very different lengths (Bidoggia et al. 2017; Gentilini et al. 2009; Pengo and Pasquato 2015; Posocco et al. 2012). The formation of separate fluorinated ligand domains could be demonstrated by electron spin resonance (ESR) and molecular simulations, and the extent of phase segregation was surprising, even when only a very small proportion of fluorinated ligands (4 %) was used (Gentilini et al. 2009; Posocco et al. 2012). In addition to ligand length mismatch and immiscibility, relative ligand ratio was found to be a third parameter affecting the morphology of the phase-segregated domains. Indeed, at intermediate ratios, “stripes” and “patches” of ligands coexisted, but as the proportion of fluorinated ligands decreased, these tended to organize into small discrete domains.

In further work, using simpler fluorinated (F) and hydrogenated carbon (H) thiols, the same group obtained a comprehensive framework of monolayer morphologies by systematically varying the structure, relative length, steric bulk, and ratio of the mixing ligands (Şologan et al. 2016a, b; Fig. 3). SAM organization was assessed through a powerful combination of atomistic/coarse-grained (CG) calculations and ^19^F nuclear magnetic resonance (NMR) experiments. In addition to ligand immiscibility and length differences, the steric hindrance and rigidity of the fluorinated chains affected the final layer morphology. Janus, patchy/striped, and random arrangements could all be identified as thermodynamic minima, so that Janus monolayers formed when H and F ligands had the same length and were at least 12 carbon atoms long (Fig. 3a). Reducing the chain to eight carbons tended to result in ill-defined morphologies (Fig. 3b), likely because the large footprint of fluorinated thiolates might favor this arrangement. For the same reason, branched ligands led to formation of random monolayers (Fig. 3c). Finally, when ligand length difference was introduced, the transition to patchy (four-carbon mismatch) or stripe-like (eight-carbon mismatch) domains was observed (Fig. 3d).

## Preparation of nanoparticles protected by patterned monolayers

The preparation of NPs displaying patterned surfaces or featuring different compartments may follow two general routes: spontaneous self-assembly of subunits or step-by-step synthesis using an external template (Du and O’Reilly 2011; Gentilini and Pasquato 2010; Reguera et al. 2013; Song and Chen 2014; Walther and Müller 2013). Regarding gold NPs, subunits used are typically thiols, given the strength of the gold–sulfur bond (~40 kcal/mol, Ulman 1996).

The first strategy exploits the fact that mixtures of dissimilar ligands can spontaneously lead to monolayer patterning due to structural mismatches, ligand immiscibility, and other constraints, as mentioned in the previous section, and there is general consensus that the resulting morphologies represent thermodynamic minima. The second strategy relies on more traditional approaches, in which monolayer patches are generated by acting directly on the NPs with external tools or by ad hoc synthetic procedures, devised to introduce the different ligands in a well-defined relative arrangement. Since metal NPs are essentially spherical and isotropic, the symmetry break necessary to achieve patterning may first require reversibly masking (protecting) a region of the surface while other regions are being functionalized, then unmasking and functionalizing it with a different ligand. The most common masking procedure is to deposit NPs onto solid supports and then generate films at air–liquid or liquid–liquid interfaces, so that two large portions of the NP surface are differentiated and can be selectively functionalized. These procedures are particularly useful for preparation of Janus surface morphologies, as from symmetry considerations multiple (sequential) patterning processes for other morphologies become increasingly complex. This technique is consequently somewhat limited in scope, and the alternative, self-assembly approach is more suited for other morphologies, provided that design principles and suitable molecular building blocks are available.

### Spontaneous patterning of the monolayer surface

**Fig. 4:**
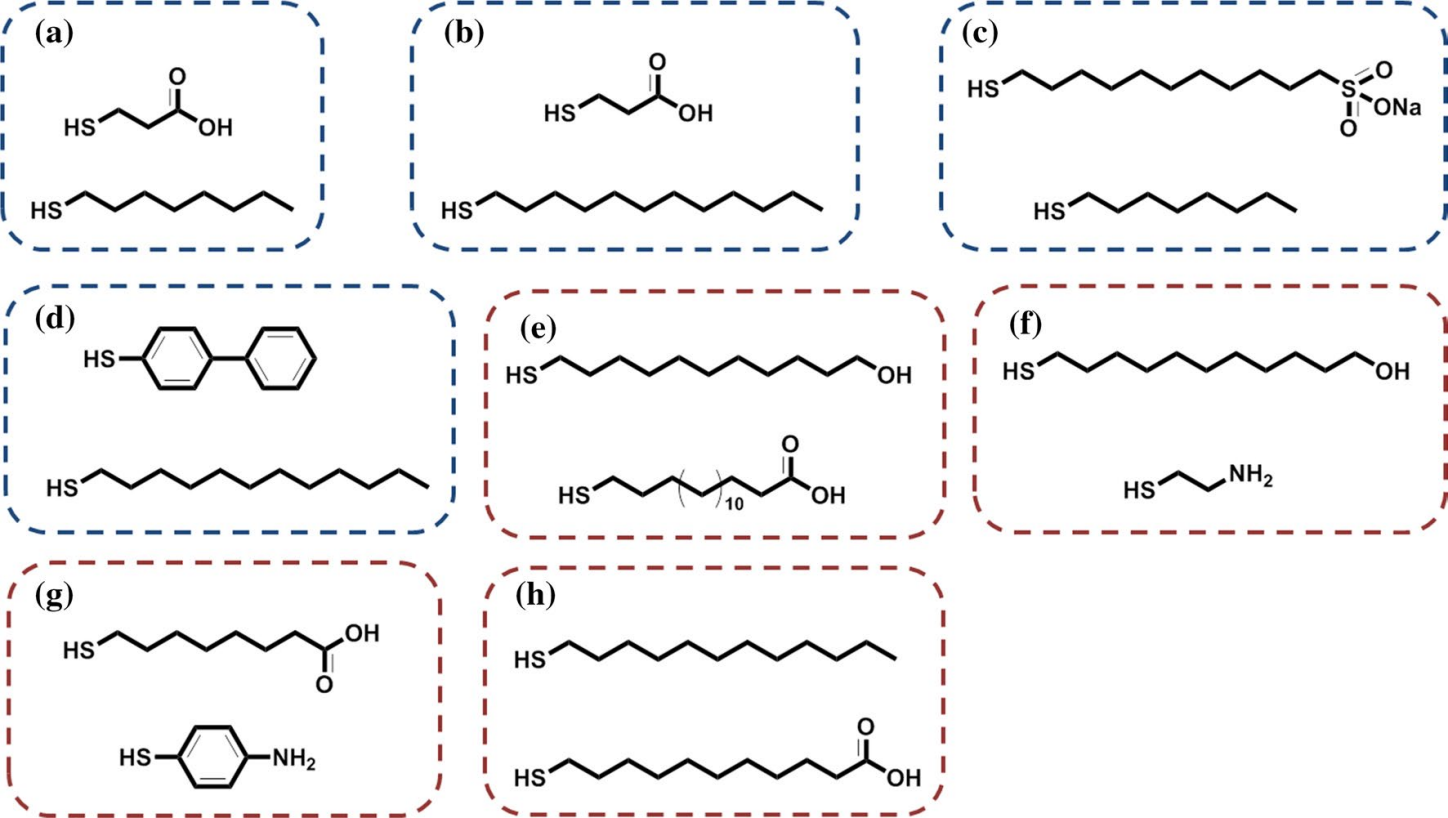
Selected thiols used for preparation of patterned monolayers via spontaneous self-assembly (**a**–**d**) or template approach (**e**–**h**). Blends of thiols (**a**–**d**) were selected for preparation of MPNPs featuring stripe-like domains (Jackson et al. 2006; Verma et al. 2008; Liu et al. 2012). Thiols (**e**–**h**) were employed for synthesis of Janus NPs with assistance of a masking surface (Sardar et al. 2007; Babajani et al. 2014; Andala et al. 2012)

From an experimental standpoint, the most well-explored MPNP systems are those in which the SAM surface is constituted by binary mixtures of ligands. Monolayer patterning by spontaneous phase segregation on metal NP surfaces was pioneered by Stellacci and coworkers (Jackson et al. 2004). They and other groups have shown that all theoretically predicted morphologies can be achieved in practice by judicious experimental design. Many ligand combinations have been explored, with the resulting surface morphologies assessed either directly or indirectly; For instance, mixtures of mercaptopropionic acid/octanethiol (OT) (Fig. 4a) [or dodecanethiol (DDT)] (Fig. 4b) (Jackson et al. 2006), 11-mercapto-1-undecanesulfonate (MUS)/OT (Fig. 4c; Verma et al. 2008), or dodecanethiol/diphenyl thiol (Fig. 4d; Liu et al. 2012), to name but a few, generate stripe-like domains; in all these cases, ligands display a significant length mismatch or other significant structural differences (aliphatic versus aromatic). The patterned NPs were obtained either by direct synthesis or by place-exchange reactions starting from preformed thiolate-protected gold NPs.

A different approach relies on the displacement of weak stabilizers adsorbed on the surface of metal NPs; in a notable example (Iida et al. 2015), patchy, random or Janus NPs were obtained by grafting amphiphilic thiols bearing oligoethylene oxide units of different lengths and terminating with either a hydroxyl group or a butyl moiety, onto the surface of 5-, 10- or 15-nm citrate-capped gold NPs. Direct, simultaneous assembly of the thiol blends resulted in patchy or random monolayers, while sequential application led mainly to Janus NPs, with the extent of phase segregation in the Janus domains being higher for 5 nm compared with 10 or 15 nm diameter. The latter evidence is in agreement with theoretical models which favor Janus-like domains for small NPs. When the core diameter decreases, the entropic interfacial gain becomes less relevant and the final monolayer morphology is determined essentially by the enthalpy of separation, which leads to spontaneous formation of completely separated distributions of ligands, i.e., Janus NPs.

An alternative approach to obtain patterned monolayers is self-assembly of suitable ligands onto NP surfaces previously stably precoated with homoligand monolayers of thiols or polymers. Spontaneous symmetry breaks were shown to occur on adsorption of lipid mixtures onto NPs precoated with either poly(allylamine)hydrochloride or 1-octadecanethiolate ligands (Yang and Murphy 2012). The NPs themselves acted as templates for the assembly of the coating lipids, with separation occurring due to charge complementarity or hydrophobic interactions and resulting in formation of separated domains for NP core diameter of 50 nm. On the other hand, when 20-nm NPs were employed, randomly mixed, homogeneous lipid layers were obtained.

Such use of intrinsically immiscible or unlike ligands to achieve phase segregation is not restricted to relatively low-molecular-weight oligomers. Blends of incompatible thiolated polymers [e.g., polystyrene/polyethylene glycol (PEG) or PEG/poly(*N*-isopropylacrylamide) (PNIPAM)] were recently reported to result in Janus-type surfaces (Percebom et al. 2016) by simple mixing with citrate-stabilized gold NPs in a common solvent. This morphology was unambiguously verified by both two-dimensional (2D) nuclear Overhauser effect spectroscopy (NOESY) and bright-field transmission electron microscopy (BF-TEM) tomography.

### Template-assisted patterning of the monolayer surface

**Fig. 5:**
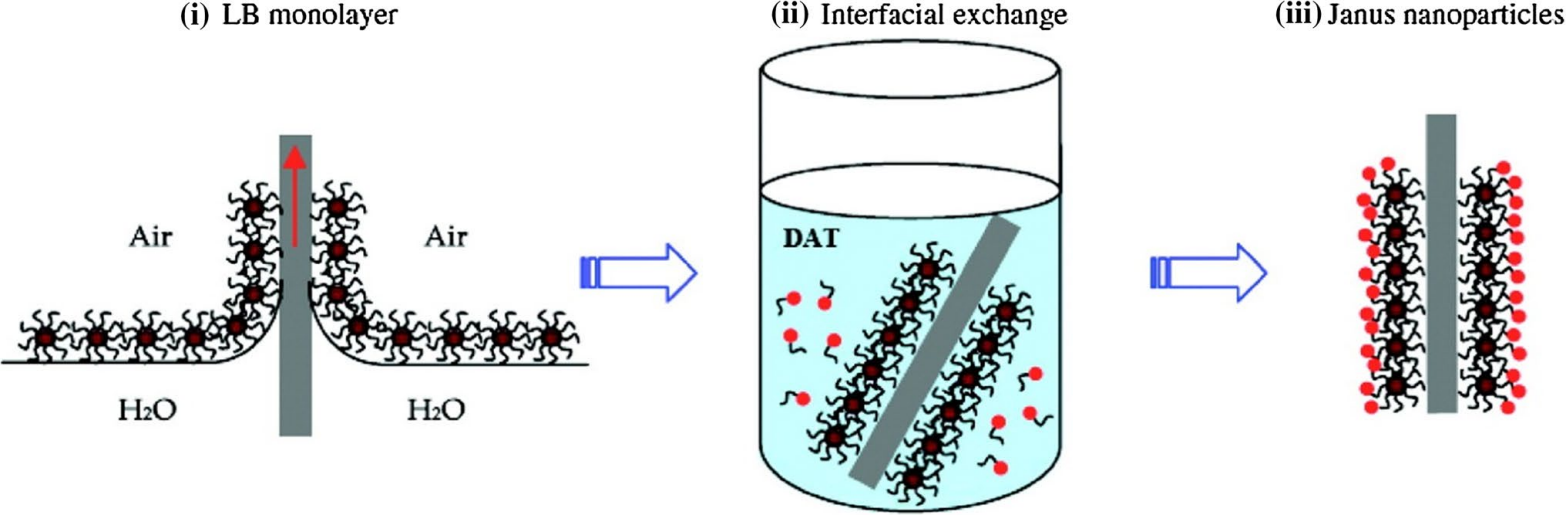
Schematic representation of Janus NP formation by interfacial exchange on a solid support. (**i**) A Langmuir monolayer is transferred to the solid support using the Langmuir–Blodgett technique; (**ii**) interfacial exchange is effected on the exposed surface using a second suitable thiol ligand; (**iii**) this results in formation of a Janus morphology on the NP surface. [Reprinted with permission from (Song et al. 2011) Copyright (2011) American Chemical Society]

Langmuir films forming at the liquid–air interface can be exploited for preparation of NPs with patterned surface monolayer, as pioneered by Chen’s group (Pradhan et al. 2007). A compact film of NPs can be induced on the surface of a solvent in which NPs are insoluble (e.g., water), by applying mechanical compression to a NP dispersion, thus bringing the particles into close contact so that half of their surface faces the solvent while the other half is exposed to air. Thiol ligands dissolved in the solvent then bind to the bottom NP surfaces, resulting in an amphiphilic Janus morphology. In a similar approach, a Langmuir monolayer of NPs treated with one thiol type was transferred to a solid support using the Langmuir–Blodgett technique (Fig. 5), and interfacial exchange effected by using a second thiol molecule (Song et al. 2011).

An alternative strategy relies on the adsorption of weakly protected gold particles onto the surface of suitable supporting materials (bulk phases or particles). An archetypal example of this method was devised by Shumaker-Parry in 2007 (Sardar et al. 2007); here, citrate-stabilized NPs (41 nm diameter) were immobilized on amino-functionalized, silanized glass surfaces and then exposed to a solution of the first thiol ligand (11-mercapto-1-undecanol) with formation of a self-assembled monolayer on the accessible NP surface. The NPs were then detached from the solid support using ultrasound and placed into a solution of the second thiol ligand (16-mercaptohexadecanoic acid or 11-mercapto-1-undecanol/mercaptoethylamine), which formed a monolayer on the previously concealed surface (Fig. 4e, f). A similar procedure was employed for preparation of 13.5-nm zwitterionic Janus-type NPs using a combination of 8-mercaptooctanoic acid and 4-mercaptophenylamine (Fig. 4g) (Babajani et al. 2014).

The need for an interface to differentiate NP surfaces does not necessarily restrict this approach to solid supports. In a solution phase approach (Andala et al. 2012), ~9-nm NPs were initially treated with dodecylamine, which binds weakly to the gold surface and allows NP solubilization in toluene. Upon transferring the NPs into a biphasic water/toluene system and addition of a 1:1 hydrophobic/hydrophilic mixture of DDT/mercaptoundecanoic acid (MUA), the functionalized NPs accumulated at the water–toluene interface (Fig. 4h). Contact angle measurements suggested Janus-like segregation of hydrophobic and hydrophilic ligands on the NP surface. A similar two-phase approach has been used to synthesize tris(hydroxymethyl)phosphine oxide/triphenylphosphine-coated Janus NPs (Luo et al. 2016).

Another interesting route for preparation of asymmetric gold NPs exploits functionalization with DNA (3′-thiolated and 5′-phosphorylated, 15-mer oligonucleotides), followed by DNA strand elongation on a single spot of the NP surface, thereby obtaining patterned monolayers (Xu et al. 2006). Magnetic microparticles bearing 30-mer oligonucleotides, with one end complementary to the oligonucleotide on the gold NP, were used to immobilize these and provide a template for elongation. This elongation was achieved by ligating another 15-mer oligonucleotide, complementary to the other end of the 30-mer, in the presence of T4 DNA ligase.

In principle, apart from its role as a masking agent for part of the NP surface, the solid support can also provide reactive moieties for NP functionalization, lamellar single crystals (12 nm thick) of thiolated polyethylene oxide being an example of such an application. This unconventional solid support was first used for anchoring 6-nm gold NPs coated with weakly bound didodecyldimethylammonium ions. The exposed NP surface was then capped with a second thiol terminating with an initiator for atom transfer radical polymerization. Poly(methyl methacrylate) and poly(*tert*-butyl acrylate) were then successfully synthesized on this portion of the NP surface, by adding the appropriate monomers. Afterwards, by dissolving the single crystal, the NP could be recovered, displaying on one side the polymeric coating and on the other the thiolated polyethylene oxide units deriving from the solid support (Wang et al. 2008).

Methodologies for monolayer patterning based on templates can reach a higher degree of complexity (Li et al. 2011; Wu et al. 2015) with respect to self-assembly approaches, as the latter result in morphologies that correspond to thermodynamic minima, which cannot be taken for granted with masking approaches. Before the NP is released from the masking particle, the ligand arrangement is kinetically stable; upon removal of the external factors responsible for phase separation, the ligands may eventually rearrange, leading to a new equilibrium morphology that may differ from the intended one (Song et al. 2011). Moreover, the ligand organization of mixed monolayers resulting from self-assembly approaches may be affected by external stimuli and may in part be induced by the presence of an external template (Boal and Rotello 2000; Norgaard et al. 2004). This phenomenon may derive from lateral diffusion of thiolates on the surface of gold NPs (Norgaard et al. 2004) and is consistent with the dynamic nature of the gold–sulfur interface (Burgi 2015; Cossaro et al. 2008; Tsao et al. 2000).

## Characterization of the structure of mixed SAMs on nanoparticles

While literature reports numerous examples of functionalized gold NPs featuring organized self-assembled monolayers, the actual structure of the ligand shell is often poorly described. Despite the importance of knowing the exact ligand shell structure as an essential prerequisite for data reproducibility and to identify predictable properties, no single straightforward experimental method for its determination is available to date, especially considering complex morphologies such as striped or patchy domains. Therefore, in this section we offer an overview of the main experimental/computational techniques commonly employed (often in combination) to assess SAM structures at molecular level, providing relevant examples, while the interested reader is referred to a recent, in-depth review on this subject (Colangelo et al. 2017).

### Experimental methods

As a general principle, information on the morphology of a mixed monolayer can only be obtained by probing its surface or domains, which can be done either directly or indirectly (Table 1). Direct probing relies on techniques such as atomic force microscopy (AFM), scanning tunneling microscopy (STM), and BF-TEM, which provide reconstructed images of monolayer structural features. Indirect methods obtain structural information from analysis of physicochemical properties of monolayer components that are expected to be influenced by the morphology itself. Alternatively, structural information can also be obtained by using molecular probes that display different properties when interacting with monolayer compartments of different nature.

**Table 1:** Summary of common experimental methods used to assess patterned SAM organization on NPs

Direct techniques	Microscopy	AFM,^a^ STM,^b^ BF-TEM^c^
	Scattering	SANS^d^
	Spectroscopy	IRRAS^e^
Indirect techniques	Spectroscopy	NMR,^f^ SERS,^g^ ESR,^h^ IR^i^
	Mass spectrometry	MALDI,^j^ IM-MS^k^

^a^Kuna et al. (2009); ^b^Biscarini et al. (2013), Centrone et al. (2007), Jackson et al. (2006), Moglianetti et al. (2014), and Ong et al. (2013, 2014); ^c^Percebom et al. (2016); ^d^Moglianetti et al. (2014); ^e^Bourone et al. (2016), Sarangi and Patnaik (2014), ^f^Guarino et al. (2012), Liu et al. (2012), Pradhan et al. (2009), and Şologan et al. (2016b); ^g^Stewart et al. (2012); ^h^Gentilini et al. (2009), Lucarini and Pasquato (2010), and Posocco et al. (2012); ^i^Centrone et al. (2007), ^j^Farrell et al. (2015), and Harkness et al. (2010); ^k^Harkness et al. (2011)

**Fig. 6:**
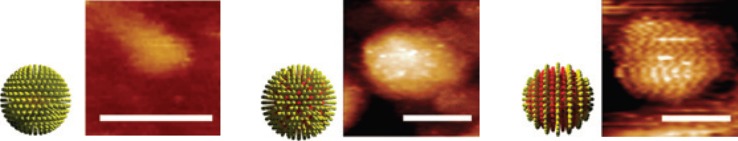
Schematic diagrams of the NP ligand shell structure next to representative STM images (scale bars 5 nm). Homogeneous monolayer (*left*), and random (*middle*) and striped (*right*) organization of hydrophilic and hydrophobic surface functional groups. [Reprinted with permission from (Verma et al. 2008). Copyright (2008) Nature Publishing Group]

Direct probing has been pursued by Stellacci’s group using STM (Biscarini et al. 2013; Centrone et al. 2007; Jackson et al. 2006; Moglianetti et al. 2014; Ong et al. 2013, 2014; Verma et al. 2008) and AFM (Kuna et al. 2009), providing the first evidence of subnanometer features on the surface of NPs compatible with the formation of well-defined phase-segregated domains (Fig. 6). While interpretation of STM data is challenging, recent improvements in STM analysis has allowed quasimolecular resolution of the NP surface (Ong et al. 2013).

In addition, analysis of the topographical power spectral density (PSD) of STM data enables determination of the characteristic length scales of monolayer features in a relatively straightforward manner (Biscarini et al. 2013), significantly facilitating interpretation of STM images. Image quality can also be improved by appropriate NP surface solvation (Moglianetti et al. 2014). In some instances, however, the interpretation of STM images of MPNPs has been debated on the basis of the very delicate instrumental settings required for such analyses (Cesbron et al. 2012; Ong and Stellacci 2015; Stirling et al. 2014). Hence, besides the technical difficulties in the interpretation of STM images, obtaining the images themselves is a complex matter. This clearly calls for alternative and independent methods, either direct or indirect, to elucidate the morphologies of mixed monolayers. In this respect, small-angle neutron scattering (SANS) has been reported to provide morphological information supporting STM analyses (Moglianetti et al. 2014), and other electron microscopy methods such as BF-TEM tomography have recently enabled reconstruction of the 3D structure of Janus-type NPs obtained by using blends of immiscible thiolated polymers (Percebom et al. 2016).

Among the indirect methods for probing monolayer structure, NMR has several advantages. In the first place, the chemical shift of nuclei (mostly ^1^H) is sensitive to their local environment, thereby reporting on the arrangement of nearby ligands which, in turn, depends on the surface morphology. Furthermore, the nuclear Overhauser effect (NOE) between vicinal chains provides information on the ligands’ reciprocal (spatial) arrangement, supporting or disproving a specific morphology. Stellacci and coworkers carried out thorough NMR analysis of mixed monolayer NPs, showing that chemical shift variations are different for random, stripe-like, and Janus-like morphologies (Liu et al. 2012). Recently, similar analysis using ^19^F NMR (one order of magnitude more sensitive to differences in chemical shift with respect to ^1^H NMR) was reported for mixed monolayer NPs coated with a hydrogenated/fluorinated ligand monolayer (Şologan et al. 2016b). The 2D NOESY experiments carried out by Pradhan et al. on mixed monolayer NPs with a random ligand arrangement showed clear cross-peaks between signals from the different ligands due to the large number of contacts arising from their intimate mixture (Pradhan et al. 2009), while these were clearly missing in the case of Janus NPs, where ligand intertype contacts are limited to domain interfaces. Stellacci’s group also observed cross-peaks for NPs with stripe-like domains. Notably, however, NMR is limited to NPs of diameter ≤5 nm, as larger sizes hinder precise ligand identification (Hong et al. 2006; Hostetler et al. 1998).

Matrix-assisted laser desorption/ionization (MALDI) mass spectrometry is another emerging method for structural characterization of mixed monolayer-decorated NPs, as the composition of low-molecular-weight fragments obtained upon laser ionization of the samples provides representative sampling of the local monolayer composition (Farrell et al. 2015; Harkness et al. 2010). Ion-mobility mass spectrometry (IM-MS) (Harkness et al. 2011) has been used to probe the mixed monolayer organization of gold NPs with binary ligand mixtures at different ratios, comparing results with theoretically derived abundance patterns from idealized monolayer models of specific morphologies. Phase separation was observed in several cases, and phase segregation was maximized by combining ligands of different length. Systems obtained by ligand exchange reactions displayed better phase separation than those obtained by direct synthesis, and in some cases it was possible to distinguish ligand domains or formation of a Janus arrangement.

In specific cases, such as for amphiphilic Janus NPs, it is possible to interrogate the individual domains of the monolayer, which however requires orienting all particles to display the same domain in one direction (for example, by using the Langmuir–Blodgett technique and transferring the film of NPs onto hydrophilic or hydrophobic supports). Alternatively, direct self-assembly on suitable substrates may be used to obtain a compact monolayer of oriented Janus NPs. These approaches have been used to characterize amphiphilic Janus NPs ranging from 3.5 to 15 nm, using infrared reflection–absorption spectroscopy (IRRAS) (Bourone et al. 2016; Sarangi and Patnaik 2014).

Molecular probes have been employed in several methodologies to study phase-segregated domains in surface monolayers (Bonomi et al. 2011). Cationic porphyrin has been used to investigate silver NPs coated with a mercaptopropanesulfonate/1-pentanethiol ligand mixture using surface-enhanced Raman scattering (SERS) (Stewart et al. 2012), while a fluorescent adenosine triphosphate (ATP) analog was used to assess the morphology of a cationic mixed monolayer on NPs using fluorescence quenching (Bonomi et al. 2011). ESR employing nitroxide radicals as probes has also been proposed (Lucarini and Pasquato 2010), the advantage of these stable radicals residing in the strong dependence of their spectral parameters on the polarity of the environment which, in turn, depends on the monolayer domains in which they preferentially reside. This provided indirect evidence of ligand phase segregation, even with domains of limited size (Gentilini et al. 2009; Posocco et al. 2012).

An alternative approach is to detect the perturbation in some property of the monolayer in a manner that can be correlated to the presence of phase-segregated domains. In this respect, Guarino et al. (2012) demonstrated how NMR signal broadening patterns induced by a paramagnetic lanthanide ion can be employed to infer the local organization of mixed monolayer NPs.

### Computational methods

Regardless of the experimental methodology employed, the resolution of NP molecular detail that can be achieved is limited and accurate determination of monolayer organization is a challenge. A comprehensive description of the structure and the organization of the monolayer can, however, greatly benefit from the coupling of experimental and computational approaches. Examples of this are the combination of SANS with ab initio Monte Carlo multiphase models (Moglianetti et al. 2014), of MALDI with self-consistent mean-field theory (SCFT) calculations (Merz et al. 2016), or of ESR or NMR measurements with multiscale simulations (Posocco et al. 2012; Şologan et al. 2016b). In this respect, the self-sorting process of ligand mixtures to form the monolayers needs to be tackled computationally either through CG techniques such as dissipative particle dynamics (DPD) (Pons-Siepermann and Glotzer 2012a, b; Singh et al. 2007) or through multiscale approaches (Posocco et al. 2012; Şologan et al. 2016b). Alternatively, due to the long times required for ligands to move on the curved surface and reach their equilibrium arrangement, statistical methods such as configurational-bias Monte Carlo can be adopted (Fetisov and Siepmann 2016; Charchar et al. 2016) (Table 2). Ligand-related properties such as molecular conformation, chain bending and tilting angles, distribution around the core, and radius of gyration can all be well described by all-atom or united-atom molecular dynamics (MD) simulations once the surface pattern has been assigned (Ge et al. 2015; Ghorai and Glotzer 2010; Heikkilä et al. 2012; Lane and Grest 2010; Van Lehn and Alexander-Katz 2013; Velachi et al. 2015, 2016). These latter techniques are also suitable for prediction of SAM interfacial properties, such as the number of interfacial solvent/ion molecules (Kuna et al. 2009; Velachi et al. 2015, 2016).

**Table 2:** Examples of computational methodologies commonly employed to model properties of patterned SAMs

Patterned SAM property	Computational technique
Self-assembly process	DPD,^a^ multiscale atomistic/CG methods,^b^ Monte Carlo^c^
Ligand structure	All-atom^d^ and united-atom^e^ MD
Interface properties	All-atom MD^f^

^a^Pons-Siepermann and Glotzer (2012a, b) and Singh et al. (2007); ^b^Posocco et al. (2012) and Şologan et al. (2016b); ^c^Fetisov and Siepmann (2016); ^d^Ghorai and Glotzer (2010), Heikkilä et al. (2012), Lane and Grest (2010), Van Lehn and Alexander-Katz (2013), and Velachi et al. (2015, 2016); ^e^Ge et al. (2015); ^f^Kuna et al. (2009) and Velachi et al. (2015, 2016)

## How patterned nanoparticles interface with biological systems

Several physicochemical and biological factors are known to govern the physiological response of engineered NPs, including the size, shape, surface chemistry, surface charge, and mechanical properties of NPs and analogous properties of cells (Gonzalez Solveyra and Szleifer 2016; Jiang et al. 2015; Oh and Park 2014), which have recently been comprehensively reviewed (Albanese et al. 2012; Beddoes et al. 2015; Mu et al. 2014). Understanding the relationships between the surface heterogeneity of MPNPs and their bioactivity is a necessary step to determine both their cell internalization and the biological responses they elicit.

### Nanoparticle interactions with membranes

The cell membrane is evidently the principal physical barrier to cellular internalization of NPs; thus, interactions between NPs and cell membranes need to be qualified and quantified using various analytical and modeling methods. Membrane models (MMs) are currently used to understand the influence of the physicochemical properties of NPs on their interactions with bilayers under controlled experimental conditions. Single interaction mechanisms such as membrane attachment, membrane disruption, or lipid property changes can be proved if MMs are used in association with the appropriate technique (Carney et al. 2013; Rascol et al. 2016; Tatur et al. 2013). Membrane attachment is mainly characterized using supported lipid bilayers (SLBs), typically associated with surface-sensitive methods such as surface plasmon resonance (SPR) (Anderluh et al. 2005; Beseničar et al. 2006), quartz crystal microbalance measurements with dissipation monitoring (QCM-D), and AFM. For AFM studies, lipids are deposited on a flat surface (mica, silicon or gold), so that alterations in the morphology of the membrane and effects such as formation of pores or holes (following NP–membrane interactions) can be observed (Morandat et al. 2013; Roiter et al. 2008). In QCM-D, on the other hand, the solid support is crystalline quartz coated with a lipid layer, and NP–SLB interactions are monitored by measuring mass changes. Both of these techniques have been applied to small, patterned NPs with diameter of 5–6 nm coated with 2:1 mixed MUS:OT monolayers; a capacity for passive insertion into the membrane was observed only in the presence of defects in the SLB (Van Lehn et al. 2014). An electrical approach (planar LB electrophysiology) was also applied to screen and quantify the interaction between surface-patterned NPs and bilayer lipid membranes (Carney et al. 2013).

Thanks to the availability of ever-increasing computing power, several theoretical studies have recently been performed to probe molecular aspects of NP–membrane interactions. The most widely applied computational techniques are classical methods such as MD, which enable exploration of structural evolution and structure–activity relationships in biological systems with atomic-level resolution (Chen and Riviere 2016). However, most biological phenomena occur on time and length scales not yet accessible to MD calculations, and more simplistic techniques such as CG (Lelimousin et al. 2016; Saunders and Voth 2013) or (almost) purely thermodynamic methods (Van Lehn and Alexander-Katz 2014c) become necessary (Rossi and Monticelli 2016).

Membranes are highly complex ensembles of different kinds of lipids and proteins, as well as carbohydrates, any of which might participate in NP interactions, and/or affect the local/global organization, fluidity, and mechanical responses. Moreover, membrane composition varies with cell type and is intrinsically asymmetric, which influences global biophysical properties such as bending rigidity and spontaneous curvature (Elani et al. 2015; Fadeel and Xue 2009), and ultimately determines the interactions with NPs. Given the difficulty in tackling such a complex biological environment, it is not surprising that the majority of computational (and MM-based) investigations employ a simplified membrane representation with the bilayer composed of one or a few different lipid molecules and devoid of proteins (Heikkilä et al. 2014; Jiaqi et al. 2010).

With respect to surface-patterned NPs interacting with membranes, some computational studies consider only Janus-type systems (Ding and Ma 2012; Gkeka et al. 2013; Ji et al. 2016; Van Lehn and Alexander-Katz 2011), while others explore how different surface patterns affect membrane interactions, and are often correlated to experimental studies. Stellacci’s group first showed that the arrangement of chemically different ligands (e.g., OT/MUS) on the NP surface impacts on their insertion pathway. Striped NPs could enter cells via spontaneous diffusion, without apparent damage to the membrane, whilst NPs with the same ligand composition but random surface distribution were internalized through the endocytic pathway (Carney et al. 2012; Sabella et al. 2014; Verma et al. 2008). A tentative explanation for this much-debated effect (Cesbron et al. 2012; Ong and Stellacci 2015) was offered by Li et al. (2012). They compared the free energy change associated with translocation through a lipid membrane for four different types of hydrophilic/hydrophobic patterned NPs, and found that a striated surface pattern restrains the rotation of the NP in the bilayer, lowering the free energy barrier to crossing the membrane, which justifies the diffusion of NPs with rippled morphology through lipid bilayers.

In an alternative explanation, the homogeneity of the ligand distribution was identified as a key parameter for enhancing NP translocation through the lipid bilayer (Gkeka et al. 2013). CG models with a standard MARTINI force field were first used to build 6-nm NPs with hydrophobic/hydrophilic implicit ligands on the surface at 1:3 ratio and then to compute the free energy pathways associated with membrane translocation and permeability coefficients. A random, heterogeneous ligand distribution resulted in hydrophobic surface clusters consisting of a few hydrophobic ligands that are not present in a perfectly homogeneous arrangement. Accordingly, groups of membrane lipids might bind to the hydrophobic clusters associated with random surface NPs, decreasing the free energy profile and favoring the interaction with the lipid bilayer core, but at the same time making the complete translocation through the membrane more difficult. A uniform surface ligand distribution instead makes passive translocation easier, as it avoids this lipid clustering at the NP surface. It is noteworthy that this explanation, while useful, needs to be further tested against more realistic, long and flexible ligands. Regarding striped topologies, the authors speculate that, if the ligand chains have enough freedom to explore the available free volume, by virtue of their flexibility they may adopt a form that more closely resembles the homogeneous surface than the random one, despite the underlying striped pattern, thus explaining the facilitated translocation.

Van Lehn and Alexander-Katz generated striped, mixed, and random MUS/OT morphologies on 2–6-nm NPs (Van Lehn and Alexander-Katz 2013). Next, they computationally modeled structural features (e.g., solvent-accessible surface area, root-mean-square fluctuations, ligand tilt angles, and radial distribution functions) in an aqueous environment at 150 mM ionic strength, at atomistic level. It was striking that quite different ligand arrangements resulted in very similar values for these parameters, suggesting that there is little possibility to distinguish the nanoscale morphology in solution. The chain length of ligands, relative to the relatively small core diameter, allows for extensive ligand fluctuations that ultimately define the properties of the NP surface more than the grafting point arrangement.

**Fig. 7:**
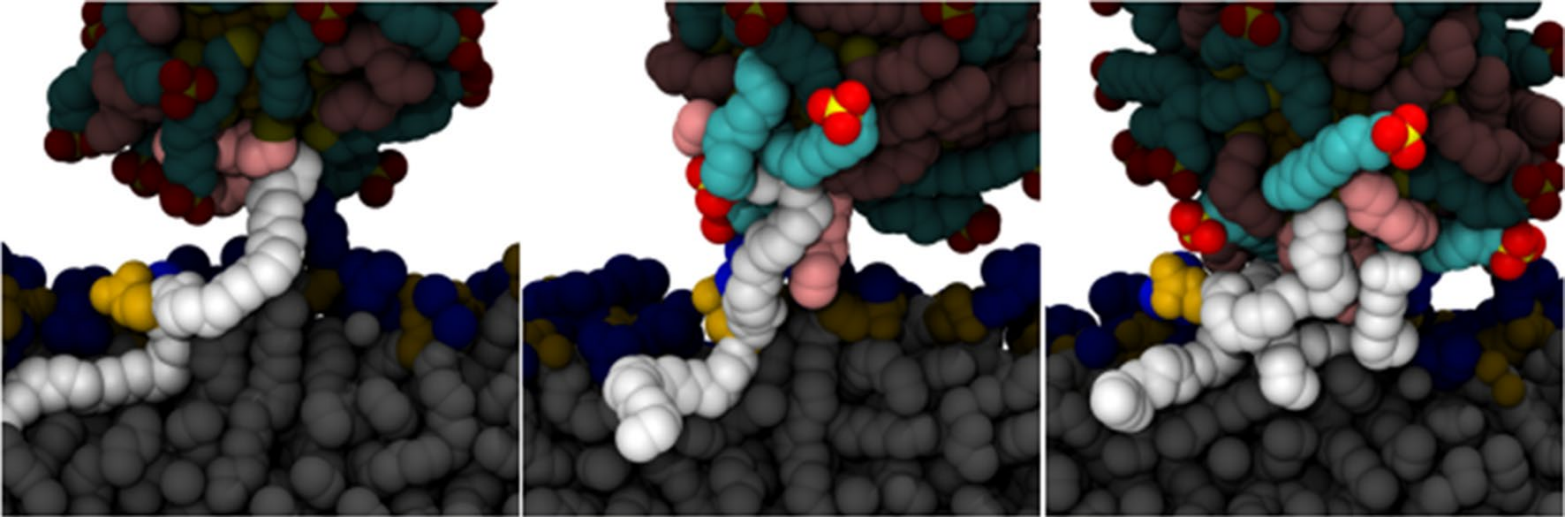
Stages of NP insertion into a membrane following protrusion contact. The first protruding membrane lipid (*left* panel), the NP ligands [MUS:OT (1:1) on a 2-nm NP] and other membrane lipids that are successively (*center* and *right* panels) recruited in forming the hydrophobic contact are highlighted in each image. Lipid tails involved in the hydrophobic contact are depicted as *white spheres*, phosphate groups are in *yellow*, and choline groups in *blue*. MUS molecules contacting the bilayer are shown as *green* (corresponding to CH_2_ groups), *yellow* (sulfur atoms), and *red* (oxygen atoms) spheres, while OT molecules are presented as *pink chains*. Lipids and ligands not presently involved in the insertion process appear dark. [Adapted with permission from (Van Lehn and Alexander-Katz 2015). Copyright (2015) Royal Society of Chemistry]

Two questions naturally arise at this stage: (1) To what extent are NPs able to *preserve* their surface ligand pattern once in contact with a lipid bilayer? and (2) How does this reflect on the effective membrane adhesion and internalization pathway of an engineered patterned material? (Lee et al. 2013). Van Lehn and Alexander-Katz provided an accurate description of the mechanism of interaction for anionic MUS/OT patterned NPs and a model 1,2-dioleolyl-*sn*-glycero-3-phosphocholine (DOPC)-composed membrane. According to those authors, the first critical step is the contact between a hydrophobic patch on the NP and a membrane lipid tail bending up and protruding into the aqueous medium. This rather rare event (Van Lehn and Alexander-Katz 2015; Van Lehn et al. 2014) represents the first energy barrier to penetration. Once this initial contact has been established, the NP monolayer ligands deform to maximize contact with the protrusion and additional membrane lipids and NP ligands are recruited into an expanding lipid/ligand mixing site (Fig. 7). The principal thermodynamic driving force for insertion is therefore the hydrophobic effect; the amphiphilic NP tries to reduce the water-exposed hydrophobic surface area by inserting into the hydrophobic bilayer core. This is accompanied by a progressively increasing membrane curvature that is relieved only when the NP core is deeply inserted into the bilayer (Van Lehn and Alexander-Katz 2015). Following the initial lipid tail protrusion event and the onset of the insertion process, one of the anionic NP ligands flips across the bilayer to the opposite leaflet, effectively irreversibly anchoring the NP, and thus initiating the fusion process.

**Fig. 8:**
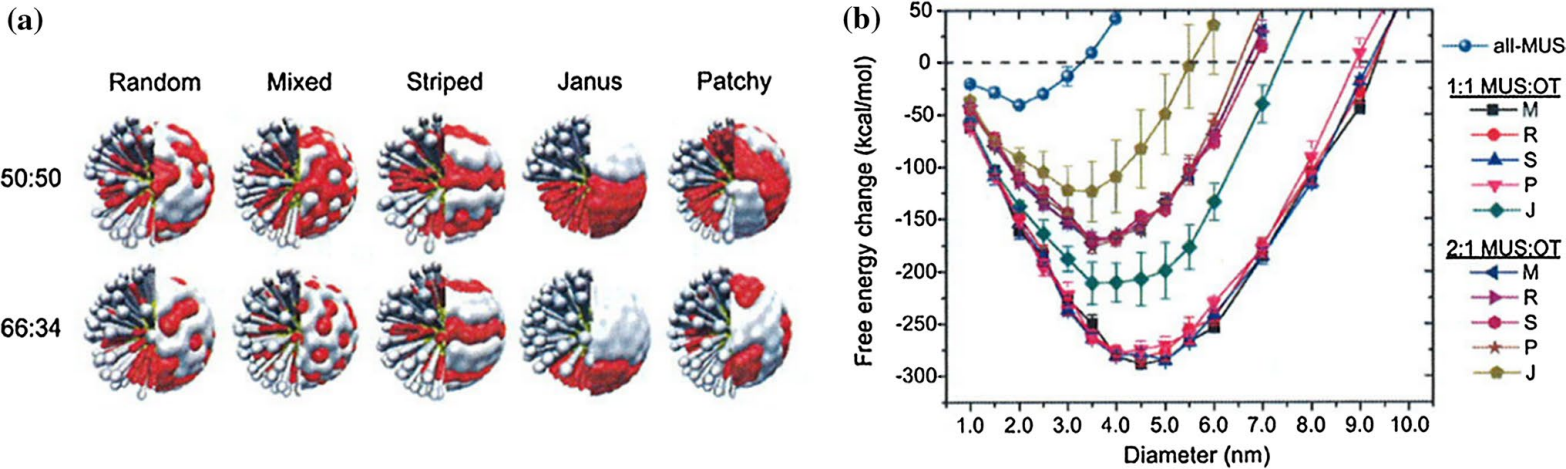
Effect of different surface morphologies and NP size on membrane insertion. **a** Representative images of different nanoscale morphologies for 1:1 and 2:1 MUS:OT surface compositions. Hydrophilic MUS ligands are depicted in *red*, while hydrophobic OT chains are represented in *white*. **b** Free energy change for insertion as a function of NP diameter for mixed (M), random (R), striped (S), patchy (P), and Janus (J) morphology for 1:1 and 2:1 MUS: OT particle. An all-MUS particle is included for reference. [Adapted with permission from (Van Lehn and Alexander-Katz 2014a). Copyright (2014) Royal Society of Chemistry]

Using an implicit solvent/implicit bilayer simulation method, it was possible to calculate the free energy change for incorporation of an amphiphilic NP into the membrane as a function of NP size and monolayer composition (Van Lehn and Alexander-Katz 2014a; Van Lehn et al. 2013). It was found to be favorable if the NP core diameter was below a defined size threshold that, however, depended on the monolayer composition. This result was confirmed by experiments on model lipid membranes (Carney et al. 2013; Van Lehn et al. 2013). Moreover, a very important clue emerged from these calculations, viz. that there is only a marginal influence of the nanoscale pattern on the free energy change associated with NP insertion (Fig. 8) (Van Lehn and Alexander-Katz 2014a; Van Lehn et al. 2013). Indeed, striped, mixed, random or patchy morphologies are virtually indistinguishable from each other for 1:1 and 2:1 MUS/OT compositions, the only exception being the Janus morphology. The monolayer composition, rather than the morphology, seems to play the dominant role in determining the likelihood of insertion. In this respect, increased ligand rigidity inhibits chain deformation and stabilization of the embedded NP through “snorkeling” (see below), especially for larger NP diameters. This affects the previously described size thresholds, shifting them to lower values (Van Lehn and Alexander-Katz 2014b).

**Fig. 9:**
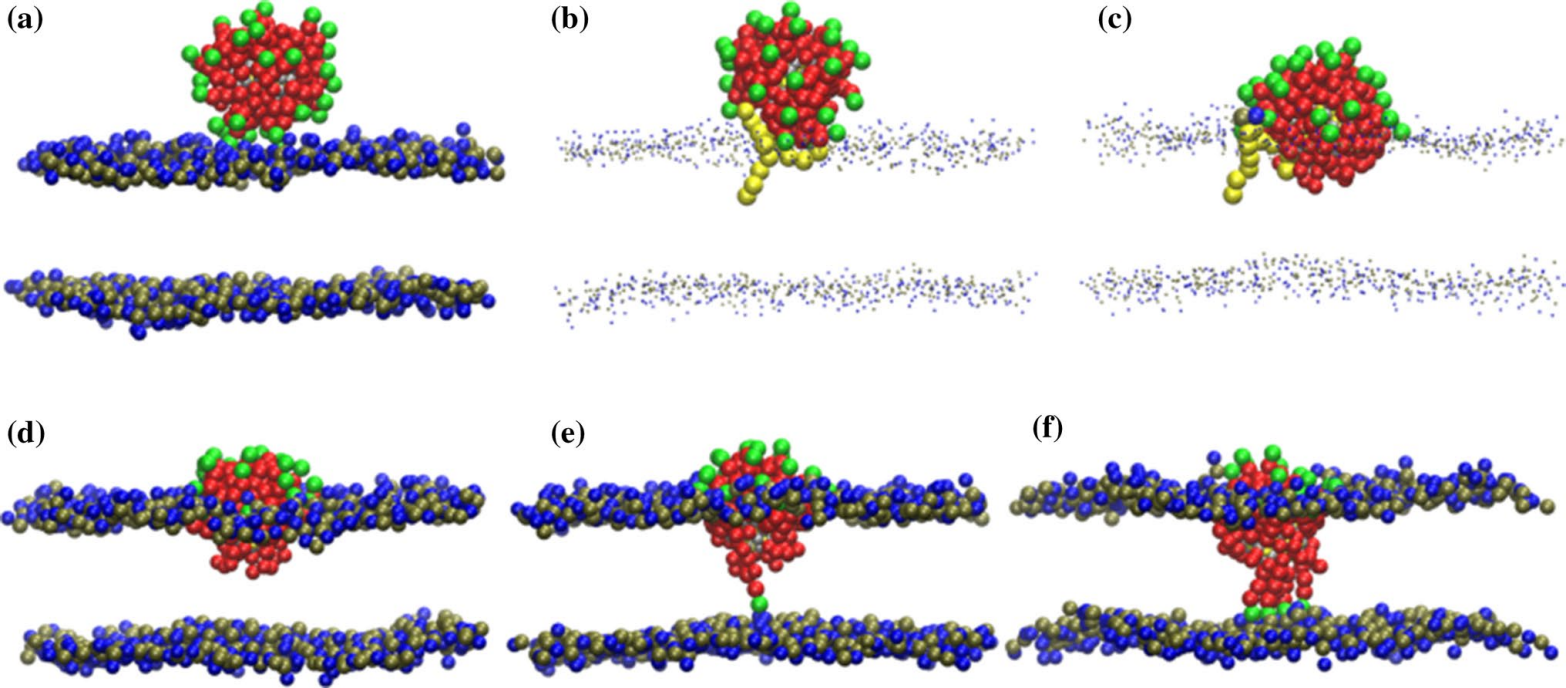
Stages of NP translocation through a biological membrane: **a** *stage 1*, adsorption of the NP at the membrane surface; **b**–**d** *stage 2*, the protrusion of a lipid tail initiates the hydrophobic contact that leads to partial embedding of the NP in the membrane core; **e**, **f** *stage 3*, the NP “snorkels” ligands to bind with the opposite leaflets (one and five anchors shown). The NP hydrophobic ligand chains are represented as *red beads*, and the charged NP ligand head-groups are represented as *green beads*. Lipid head-groups (choline) are shown as *blue beads*, while *tan beads* represent phospholipid phosphate groups. Water molecules and membrane phospholipid tails are not shown, except in **b** and **c**, where only the hydrophobic tails of the protruding lipid are represented by *yellow beads*. All snapshots refer to a MUS:OT 1:1 random NP. [Reproduced with permission from (Simonelli et al. 2015). Copyright (2015) American Chemical Society]

On the other hand, experimental evidence suggests that MUS:OT NPs featuring alternating hydrophilic/hydrophobic stripes are internalized significantly more efficiently than those with the same ligand composition but random organization (Carney et al. 2012; Sabella et al. 2014; Verma et al. 2008). Simonelli et al. (2015) coupled faster unbiased CG simulations (suitable to capture the *kinetics* of NP insertion over longer time and length scales) with biased free energy calculations (able to shed light on the *thermodynamics* of the translocation); their results, consistent with the general picture emerging from biased atomistic calculations (Van Lehn and Alexander-Katz 2015), identified three main stages in anionic patterned NP insertion (Fig. 9):

1. *Adsorption at the membrane surface* This step involves electrostatic-driven adhesion to the head region of the membrane. The time the NP spends at this interface is in the order of microseconds and is *not* influenced by the ligand arrangement, which conversely affects the strength of interaction with the lipid heads, so that 1:1 MUS:OT striped NPs outperform random NPs (both 1:1 or 2:1). Unlike patched NPs, which never detach from the membrane surface once adhered, random NPs were observed to occasionally dissociate from the upper leaflet, suggesting less stable and optimized binding to the lipid heads.
2. *Formation of a hydrophobic contact* The second interaction stage is initiated by the protrusion of one lipid tail to the head region (with an energetic cost in the range of 4–11 *k*_B_ *T*). Once the protrusion has triggered the formation of a hydrophobic contact, the NP ligands rearrange in such a way that the hydrophobic moieties contact the membrane hydrophobic core and the negatively charged ligands contact the choline groups of the lipids. The typical lifetime of this stage is in the order of nanoseconds for random NPs and microseconds for patched NPs.
3. *Step*-*by*-*step progression to the snorkeling configuration* During the last stage, the NP stabilizes its position within the membrane core, consecutively flipping ligand chains with charged terminal groups through the bilayer and contacting the lipid head-groups of the opposite leaflet, leading to the so-called snorkeling configuration.

Moreover, the free energy profile for membrane insertion of NPs with a random particle surface arrangement presents two metastable minima, one corresponding to the membrane surface-adsorbed configuration (Fig. 9b) and the other to the snorkeling configuration (Fig. 9e, f). If the membrane surface ligands are not randomly arranged but assemble in patches, the NP goes through three metastable configurations. In this case, the third state corresponds to the formation of a stable hydrophobic contact between the NP and lipid heads (semiadsorbed state) (Fig. 9d). As a consequence, patched NPs have to overcome an additional energetic barrier to cross the hydrophobic core of the membrane and anchor to the opposite leaflet, increasing the time required for translocation. This might explain why the average lifetime for stage 2 in the case of random NPs is three orders of magnitude shorter than for patched NPs.

These computational results reveal a significant role of ligand arrangement in the kinetics and thermodynamics of the interaction of patterned NPs with membranes. Unfortunately, they do not completely clarify the experimental evidence indicating that NPs featuring striated domains can passively penetrate the cell without toxic effects, while NPs with the same ligand ratio but lacking order are internalized through an endocytic pathway (Carney et al. 2012; Sabella et al. 2014; Verma et al. 2008). One should also remember that the molecular models of random and patched NPs were artificially constructed by mixtures of MUS and OT ligands, whilst random NPs were experimentally obtained using MUS and *branched* OT chains. As alternative scenarios, in a more realistically crowded membrane environment, the different kinetics of bilayer interactions with random and patched NPs might affect different types of interactions of the NPs with other membrane constituents or membrane-embedded proteins, eventually leading to quite different translocation pathways, as proposed by Simonelli et al. (2015). In addition, the possibility of cooperative effects arising from NP self-association (either after adsorption to the membrane surface or after embedding into the membrane core) also needs to be taken into account; yet, this aspect is still poorly investigated at the computational level due to the difficulties in sampling the long time and length scales involved (Alexander Alexeev et al. 2008; Gkeka et al. 2013; Li et al. 2014b). In this respect, multiple mixed ligand anionic NPs, when *inserted* into the membrane core, show a remarkable similarity in behavior to membrane-embedded proteins (Angelikopoulos et al. 2017); among other effects, they increase the probability of lipid protrusion, suggesting that the energy barrier for anchoring could indeed be decreased due to cooperative effects between particles (Van Lehn and Alexander-Katz 2014c). In addition, embedded NPs induce local bilayer thinning, a phenomenon also observed for proteins, being shown to drive aggregation, implying that NPs may also experience such membrane-mediated attraction.

Overall, an unambiguous and comprehensive understanding of the mechanism of interaction of patterned gold NPs with the cellular membrane is still lacking at present, and the number of pertinent studies, computational and experimental, quite limited. Theoretical investigations are performed with different methodologies, each having its own inherent peculiarities and limitations. This may explain why independent studies may lead to apparently contrasting results, and why it is at present difficult to establish a general framework of understanding. Furthermore, in silico analysis relies on simplified models that complicate direct comparison with experiments. As a consequence, there is an urgent need to fill this gap with new experiments designed ad hoc. Furthermore, in the majority of the computational studies, all monolayer morphologies (be they stripes, patches or Janus) and related properties (both structural and/or relating to the interaction with membranes and proteins) are simulated using the same mixture of ligands and imposing the desired pattern. This, obviously, does not fully correspond to the real monolayer morphology. Moreover, how the mobility and stability of the ligands are affected when in contact with biological molecules is even harder to establish at present.

### Nanoparticle interactions with proteins

When NPs are exposed to biofluids such as plasma and serum, proteins (as well as other biomolecules) may be dynamically adsorbed onto their surface, forming a so-called protein corona (Monopoli et al. 2012; Walkey and Chan 2012). This alters the size, aggregation state, and interfacial properties of the nanomaterial, endowing it with a biological identity that is quite distinct from its native synthetic one (Docter et al. 2015). It is this biological identity that elicits physiological responses (namely agglomeration, cellular uptake, circulation lifetime, signaling, kinetics, accumulation, and toxicity) by mediating the interaction of the nanomaterial with biomolecules, membranes, and physical barriers (Setyawati et al. 2015).

The precise mechanism(s) of formation of the protein corona is still far from being adequately explained, since it is a complex process that depends on numerous parameters pertaining to the nanomaterial (size, shape, charge, hydrophobicity, composition, surface functionalization, and topography), the proteins (size, shape, charge, surface functionality, isoelectric point, and conformational flexibility), the physiological environment (polarity, ionic strength, pH, and temperature), and the exposure time (Mahmoudi et al. 2011, 2013; Shemetov et al. 2012).

The use of targeted NPs in bio-nanotechnology could therefore be improved by manipulating their surface properties to bind proteins selectively in order to modulate or control effects on signaling, uptake kinetics, transport, accumulation, and toxicity (Mahmoudi et al. 2011; Schick et al. 2014). Meanwhile, the NP itself may alter the structure of the adsorbed proteins, leading to significant conformational changes—up to denaturation—with concomitant loss of their biological function and potentially hazardous consequences (Chen et al. 2014; Kim et al. 2013; Kopp et al. 2017; Saptarshi et al. 2013; Wang et al. 2017).

Despite the ever-increasing number of studies dedicated to uncovering the detailed relationships between synthetic and biological identities, and physiological responses to nanomaterial–protein complexes (Albanese et al. 2012; Tenzer et al. 2013; Walczyk et al. 2010; Walkey and Chan 2012), a comprehensive description is missing due to the inherent complexity of physiological systems (Pino et al. 2014) and to the experimental difficulty of defining the characteristics of the corona without altering its original nature while doing so. To further complicate matters, most present studies involve NPs with homogeneous surfaces while only a few have consider the influence of surface heterogeneity at the nanoscale (in a comparable size range to proteins).

Proteomic analyses have used liquid chromatography–mass spectrometry (LC–MS) to probe the composition of the corona, and showed that it may not correlate simply with plasma protein abundance, or their size/charge (Tenzer et al. 2011). Furthermore, some studies on patterned NPs have shown that the surface ligand composition and morphology affect how proteins such as bovine serum albumin (BSA) bind. In fact, fluorescence quenching, dynamic light scattering (DLS), circular dichroism (CD), and isothermal titration calorimetry (ITC) performed with either striped or randomly distributed polar/nonpolar groups on NPs suggest different “side-on” or “end-on” BSA conformations on the NP, depending on its shell organization. For a surface with randomly distributed ligands, binding is mainly mediated by electrostatic interactions, as charged groups are uniformly distributed on the NP surface, while for a striped surface, a combination of different interactions come into play, due to the presence of both polar and apolar groups in well-defined striations. BSA may thus adjust its binding conformation to optimize interactions with NPs presenting different types of surface (Huang et al. 2014).

**Fig. 10:**
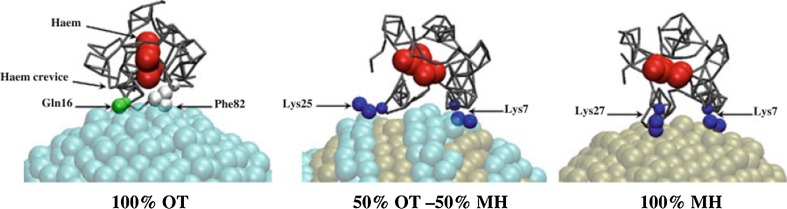
Putative binding conformations for cyt *c*–MH/OT NPs. Equilibrated structures were obtained from CG calculations with varying MH-to-OT ratio. Tightly bound residues (*small spheres*) and heme (*large spheres*) are highlighted in each cyt *c*–NP complex. [Reprinted with permission from (Hung et al. 2011). Copyright (2011) American Chemical Society]

In another combined experimental/computational investigation, interactions of cytochrome *c* (cyt *c*) with nanostructured surfaces formed with mixtures of 6-mercapto-1-hexanol (MH) and OT ligands were explored using protein assays and computational MD simulations (Hung et al. 2011). The colorimetric microBCA (bicinchoninic acid) protein quantification assay and calculated binding enthalpies highlighted that the protein exhibited increased adsorption with an increased MH proportion, suggesting that cyt *c*–surface interactions are largely hydrophilic. The amphiphilic lysine side chains of cyt *c* were able to closely contact both polar and nonpolar surface ligands simultaneously, and NPs that exhibited such nanoscale chemical domains adsorbed the protein with a specific geometrical conformation (Fig. 10).

The same key role of the surface structural and chemical heterogeneity of nanoscale patterned NPs was also confirmed by a further computational study involving lysozyme and specifically patterned planar surfaces of self-organized MH and OT thiols (Hung et al. 2013). A number of different amphiphilic amino acids, including tyrosine and tryptophan, were found to interact with NP surfaces via water-mediated contacts. Bridging water molecules adopt orientations different from those of simple, surface-adsorbed waters, facilitating protein–surface contacts.

In any case, an intimate NP–protein interaction requires biomolecules to possess size and surface properties (e.g., hydrophilic/hydrophobic patches) complementary to those present on the patterned NP surface. In other words, the interaction between proteins and NPs is determined by the surface heterogeneity of the NPs, but also depends on the scale of protein heterogeneity as well as its size (Huang et al. 2013).

Taken together, these findings suggest not only that nanopatterned surfaces can be designed to selectively combine with different proteins, but that proteins may also be engineered to specifically interact with nanomaterials by targeted incorporation of amphiphilic amino acids (natural or synthetic) possessing multiple affinities to different chemical motifs.

### Nanoparticle interactions with cells and biological systems

Interactions of NPs with biological membranes and their subsequent internalization within cells represent fundamental steps to exert their bioeffects. Even though NPs are often described as “safe,” their retention within the organism could result in toxic effects, and in fact NPs have been reported to trigger oxidative stress, inflammation, and DNA damage. The effects are particularly relevant in case of retention at high concentrations for long periods, or even permanent retention inside cells and tissues of organs such as liver and spleen, which could eventually be compromised (Frohlich 2012; Sabella et al. 2014). The extensive literature on these aspects of the biological activity of NPs has recently been reviewed by Fratoddi et al. (2015), who also summarized the assays most commonly used to determine effects of NP exposure on cell viability. These include (1) membrane damage assays such as uptake of dyes (e.g., trypan blue or propidium iodide, PI) or cellular release of calcein or lactate dehydrogenase, (2) viability tests such as the 3-(4,5-dimethylthiazol-2-yl)-2,5-diphenyltetrazolium bromide (MTT) or 2-(4-iodophenyl)-3-(4-nitrophenyl)-5-(2,4-disulfophenyl)-2*H*-tetrazolium (WST-1) assays, or (3) measurements of fluorescent substrates reporting on apoptotic cell damage. Unfortunately, the correlation between the physicochemical parameters of NPs and their overall toxicity as measured by these assays is not straightforward (Henriksen-Lacey et al. 2017), and standardization methods are required to assess the overall dose-dependent toxicity in cultured cells and allow a more robust correlation. Further critical points that are worth considering are (a) toxic effects are usually reported relative to the mass/volume of NPs, while it is more likely to be associated with the available surface area, and (b) monodispersion or aggregation of NPs is quite likely to influence their capacity to enter cells and thus regulate their bioeffects and/or toxicity.

A wide variety of cell models have been used to test the effects of NPs, but there is a general consensus that cells should be in the logarithmic growth phase, when they are more sensitive to toxic effects than in the stationary phase (Treuel et al. 2013). This being said, appropriate coating of NPs not only affects the mode and efficiency of internalization (Carney et al. 2012; Gong et al. 2015; Mukhopadhyay et al. 2012; Saha et al. 2013; Van Lehn et al. 2013; Verma et al. 2008) but may also attenuate potential toxic effects (Oh et al. 2011; Ritz et al. 2015). Interestingly, NPs endowed with similar overall surface compositions but different surface morphologies (e.g., striped versus random ligand arrangements) can have similar internalization capacities into cells, but use quite different internalization mechanisms, and have correspondingly different cell toxicities.

**Fig. 11:**
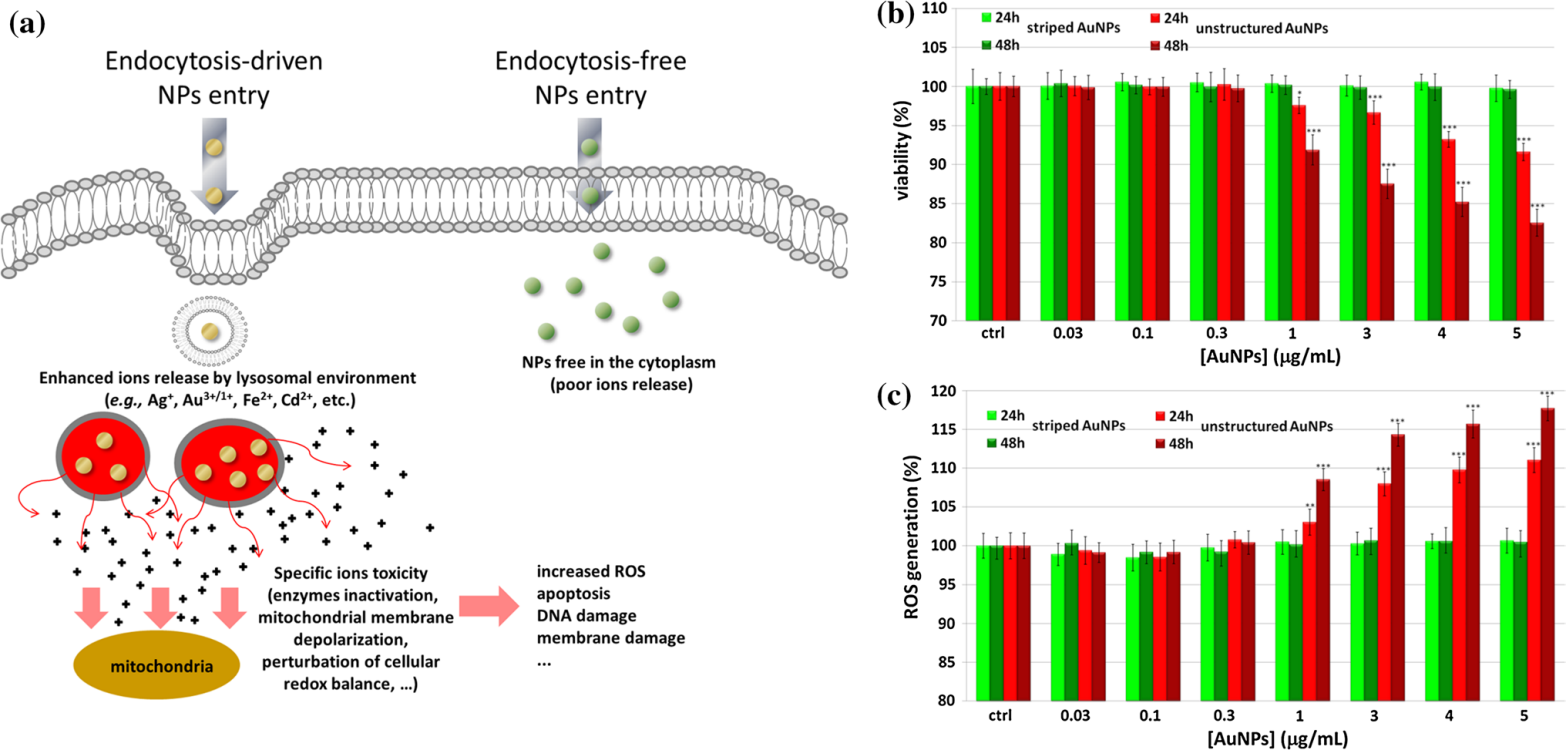
NP toxicity in relation to internalization mechanism. **a** NPs entering the cells by energy-dependent processes (e.g., clathrin, caveolin, or lipid raft-related endocytosis) are directed via endosomes to lysosomes, where the acidic pH triggers the LETH effect, with enhanced release of toxic ions (e.g., Au^1+/3+^). This effect is less important for freely diffusing NPs. **b** Correlation between AuNP surface morphology and cell viability. **c** Correlation between morphology and production of toxic reactive oxygen species (ROS). [Adapted from (Sabella et al. 2014). Published by The Royal Society of Chemistry]

In particular, it has been reported that NPs with random morphologies are more toxic than striped ones. Toxicity can depend on the entry mechanism, and in this respect, a fundamental role has been ascribed to lysosome, due to the so-called lysosome-enhanced Trojan horse (LETH) effect (Sabella et al. 2014) (Fig. 11), by which NPs internalized via endocytic vesicles undergo acid lysosomal degradation that results in release of ions that are toxic to cells. This does not occur for NPs that traverse the plasma membrane by passive diffusion and therefore avoid lysosomal inclusion.

**Fig. 12:**
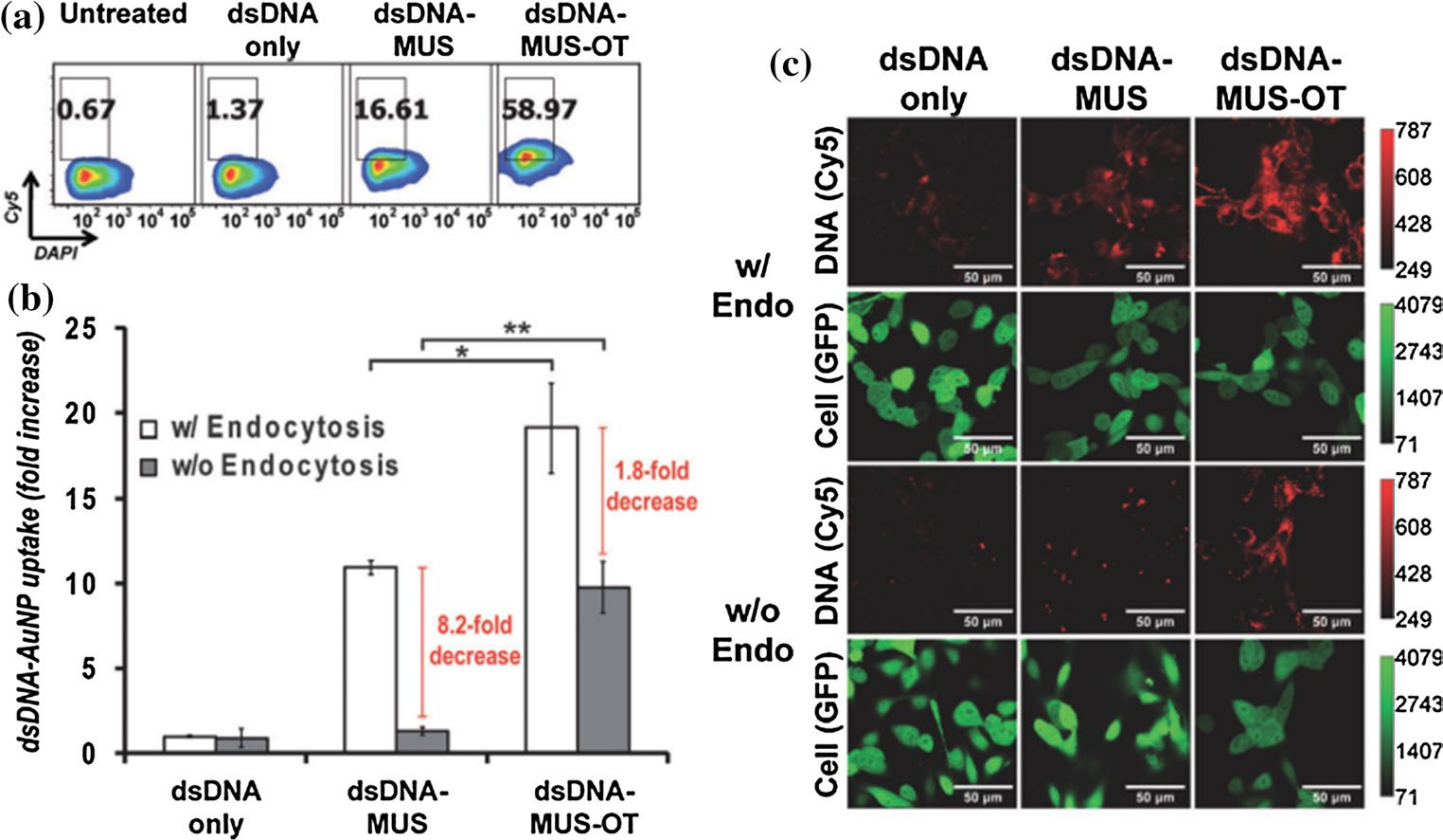
Ligand-functionalized AuNPs mediate efficient dsDNA delivery to cells through ligand structure-dependent entry mechanisms. B16-F0 melanoma cells were incubated for 4 h in serum-free medium with free Cy5-labeled dsDNA or Cy5-DNA-functionalized AuNPs. **a** Flow cytometry histograms demonstrating uptake of DNA and cell viability [4′,6-diamidino-2-phenylindole (DAPI)] under normal cell culture conditions. **b** Relative frequency of dsDNA^+^ cells (normalized to frequency of cells taking up free dsDNA under endocytic conditions), assessed by flow cytometry in presence (*white bars*) or absence (*grey bars*) of endocytosis (*p < 0.05; **p < 0.01). **c** Confocal laser scanning microscopy analysis of AuNP-mediated delivery of fluorescent DNA (*red channel*) to B16-F0 melanoma cells expressing green fluorescent protein (GFP, *green channel*) as a cytosolic/nuclear marker. [Reprinted with permission from (Jewell et al. 2011). Copyright (2011) Wiley]

Drug delivery is one of the main fields of bioapplication for patterned NPs (Atukorale et al. 2015). Striped MUS/OT NPs were able to transport single- and double-strand DNA of various lengths into B16-F0 melanoma cells with enhanced efficiency with respect to free DNA and homoligand MUS NP-mediated delivery and without significant toxicity associated with the cargo uptake (Fig. 12). Another major point emerging from this study is that AuNPs coated with ribbon domains preserved the entry mechanism observed in the absence of drug even when conjugated with more than one oligonucleotide (Jewell et al. 2011). The same amphiphilic ligand-coated AuNPs also exhibited remarkable lymph node tissue accumulation with preferential uptake in myeloid dendritic cells. When tested for vaccine delivery after conjugation with a peptide antigen (SIINFEKL), they drastically improved the peptide vaccine response compared with free antigen or linker-antigen administration and effectively protected against tumor outgrowth (Yang et al. 2017).

In combination with other additives, these engineered NPs were also found to improve the thermal stability of viral formulations in vaccines, replicating the stabilizing effect of sucrose but at much lower concentrations. Even if not strictly related to the NP nanoscale surface heterogeneity, the formation of a cloud of negatively charged NPs around the virus particles favors confinement of the DNA within the capsid and alters the capsid rupture limit, thus enhancing virus lifetime (Pelliccia et al. 2016).

Several examples of metallic nanomaterials employed as adjuvants to radiotherapy have been reported due to their strong interaction with ionizing radiation. Exploiting the ability of amphiphilic stripe-like AuNPs to penetrate membranes, MUS/OT NPs were loaded into multilamellar lipid vesicles and delivered to tumor cells, where they increased the cell killing ability relative to irradiation alone and free NP uptake, albeit in a cell-dependent manner (Yang et al. 2014).

## Conclusions

Controlling self-assembly of complex materials using a bottom-up approach is a key theme in nanotechnology. Self-organization of small ligands at the surface of metal nanoparticles represents a versatile starting point for preparation of (bio)nanomaterials with precise (bio)physical and (bio)chemical properties. Directing nanoparticle morphology creates surfaces with specific energetics and chemical reactivity, which can be employed to regulate assembly and ligand presentation or spatial arrangement on the nanoparticle surface. In turn, nanomaterials with precise surface organization may be tailored to selectively interact with proteins or other biological targets. Controlling interactions with this level of specificity enhances our capacity to control how engineered nanomaterials negotiate with biological systems at molecular level.

Thanks to recent developments in nanoparticle synthesis and better understanding and control over surface chemistry, there have been an increasing number of reports on nanoparticles protected by self-assembled monolayers with specific patterns. However, establishing a direct correlation between surface decoration and biological effect remains a major challenge. A high level of integration between theoretical and experimental methodologies may help to fill the knowledge gap and establish a comprehensive framework to describe this correlation in detail. Advances in both of these areas are needed to overcome current limitations, and it is our hope that this review can contribute to the identification of the design rules and characterization approaches that are required for precise control over nanomaterials with reliable, desired biological responses.

## Abbreviations

AuNP: Gold nanoparticle
AFM: Atomic force microscopy
ATP: Adenosine triphosphate
BCA: Bicinchoninic acid
BF-TEM: Bright-field transmission electron microscopy
BSA: Bovine serum albumin
CD: Circular dichroism
CG: Coarse-grained
Cyt *c*: Cytochrome *c*
DAPI: 4′,6-Diamidino-2-phenylindole
DDT: Dodecanethiol
DLS: Dynamic light scattering
DOPC: 1,2-Dioleolyl-*sn*-glycero-3-phosphocholine
DPD: Dissipative particle dynamics
ESR: Electron spin resonance
IM-MS: Ion-mobility mass spectroscopy
IR: Infrared spectroscopy
IRRAS: Infrared reflection–absorption spectroscopy
ITC: Isothermal titration calorimetry
LETH: Lysosome-enhanced Trojan horse
LC–MS: Liquid chromatography–mass spectrometry
MALDI: Matrix-assisted laser desorption/ionization
MD: Molecular dynamics
MH: 6-Mercapto-1-hexanol
MM: Membrane model
MPNP: Monolayer-protected nanoparticle
MTT: 3-(4,5-Dimethylthiazol-2-yl)-2,5-diphenyltetrazolium bromide
MUA: Mercaptoundecanoic acid
MUS: Mercaptoundecanesulfonate
NMR: Nuclear magnetic resonance
NM: Nanomaterial
NOE: Nuclear Overhauser effect
NOESY: Nuclear Overhauser effect spectroscopy
PEG: Polyethylene glycol
PNIPAM: Poly(*N*-isopropylacrylamide)
PI: Propidium iodide
PSD: Power spectral density
OT: Octanethiol
QCM-D: Quartz crystal microbalance with dissipation monitoring
ROS: Reactive oxygen species
SAM: Self-assembled monolayer
SANS: Small-angle neutron scattering
SCFT: Self-consistent mean-field theory
SERS: Surface-enhanced Raman scattering
SLB: Supported lipid bilayer
SPR: Surface plasmon resonance
STM: Scanning tunneling microscopy
WST: 2-(4-Iodophenyl)-3-(4-nitrophenyl)-5-(2,4-disulfophenyl)-2*H*-tetrazolium
